# Surgery

**Published:** 1861-01

**Authors:** S. W. Gross

**Affiliations:** Lecturer on Surgical Anatomy and Operative Surgery, Philadelphia


### Bi-Monthly Abstracts;

OR, BI-MONTHLY NOVELTIES IN MEDICAL, PATHOLOGICAL, SURGICAL, OBSTETRICAL PHYSIOLOGICAL, AND CHEMICAL SCIENCE.

### SURGERY.

BY S. W. GROSS, M.D.,

LECTURER ON SURGICAL ANATOMY AND OPERATIVE SURGERY, PHILADELPHIA.

### I.—*On the Treatment of Aneurism by Digital Compression.*

WE have from time to time recorded all the cases of aneurism treated by digital compression, which have fallen under our notice, our desire having been to place the subject prominently before our readers, in order that they might draw their own conclusions in regard to the value of a plan of treatment, which is, undoubtedly, one of the most expeditious and effective modes of bringing about a cure. We have now to add six new cases to those already reported, three being traumatic, and affecting the palm of the hand, two popliteal, and one aneurismal enlargement of the thyroid gland.

1. In the *Oesterreichische Zeitschrift für praktische Heilkunde*, No. 27, 1860, Dr. Winkelhofer describes a case of traumatic aneurism of the superficial volar artery, of the size of a pigeon's egg, and of fifteen days' standing. The cutaneous wound had not united, and presented a gangrenous appearance; the arm was œdematous, and the general condition of the patient was bad. The compression was made on the brachial artery, in an intermittent manner, for several hours every day. From the second day the pulsation became weaker, and had entirely disappeared at the expiration of eight days, when the tumor was hard and painless, and had diminished two-thirds. Eight weeks after the accident there was no trace of the tumor.—(*Gazette Hebdomadaire*, vol. vii. No. 39, 1860.)

2. A year since, M. Verneuil reported to the Surgical Society of Paris a case of traumatic aneurism of the ulnar artery in the palm of the hand, which was cured by digital compression on the brachial artery. We are unable to give any further details of this case.—(*Ibid.*)

3. Professor Herrgott communicated to the Medical Society of Strasbourg, in June last, the history of a case, which may be found in the *Gazette Médicale de Strasbourg*, No. 8, 1860, of false aneurism of the right superficial palmar arch, in which digital compression having proved unavailing, a cure was effected by the ligature. The examples of Drs. Winkelhofer and Verneuil show, however, that compression may be followed by a successful result.—(*Ibid.*)

4. The *Oesterreichische Zeitschrift für praktische Heilkunde*, page 25, 1860, contains an account of an interesting case of popliteal aneurism, under the care of Dr. Giacich, in which digital compression having been made by assistants, without success, the patient succeeded in effecting a cure with his own fingers. The subject was thirty-nine years of age, and perceived the tumor in the right popliteal space two months previous to his admission into the Fiume Hospital. The digital compression was maintained from twelve to fifteen hours each day. At the end of eight days, the aneurism having undergone no change, and the circulation being accelerated, the patient was bled, digitalis was administered, and cold applied to the tumor. In the intervals of compression by means of the finger, Signorini's tourniquet was applied over the femoral artery. In a fortnight, no material modification having been effected, although the intolerable pain in the ham had ceased, the ligature of the artery was proposed, to which the man would not submit. He left the hospital and resumed his usual occupation, but being attacked again with great pain, he took to his bed, and tried digital compression for about two hours and a half every day.

After having continued this treatment for some time, he again presented himself to Dr. Giacich, who found that the tumor was perfectly obliterated and much diminished in size. The patient had made the compression for sixty-six days, of which fifteen were passed in bed, and it was kept up for one hundred and sixty-five hours. When we add to this the time he spent in the hospital, we find that compression had been exercised during eighty days, for two hundred and forty-seven hours.—(*Ibid.*)

A case somewhat similar to the above was reported by Mr. Colles, in 1854, in which the patient cured himself, without assistance, of a diffuse popliteal aneurism, by intermittent digital compression, in seven days.

5. In a communication made to the Medical Society of Constantinople, Dr. Brunelli related the history of the case of a woman, who, by making digital compression over the common carotid, had succeeded in relieving herself of an aneurismal goitre. The tumor was as large as an infant's head, and the superior thyroid artery coursed over its upper portion, becoming lost in the gland itself. On puncturing the mass, arterial blood flowed out in a jet, and the tumor almost immediately refilled. Dr. Brunelli taught the patient the mode of compressing the carotid, which was difficult, on account of its being covered by the tumor, rendering it necessary to elevate it and carry the finger very deeply back. The woman compressed alternately the carotid and superior thyroid. At the expiration of five months the tumor had lost its elasticity, was hard and not more than half as large as a hen's egg. Seeing the swelling so much reduced she abandoned the treatment, and no relapse has occurred.—(*Gazette Médicale D'Orient*, No. 5, 1860.—*Ibid.*)

6. Early in November, 1860, Professor Gross had, at the clinic of the Jefferson Medical College, a negro man, thirty-eight years of age, who discovered a small pulsating tumor in the left popliteal space two years previously, which had increased so much in size as to cause the leg to be immovably flexed, and be the seat of severe pain. The knee of the affected side measured seven inches more in circumference than the sound one, and the signs of aneurism were well marked.

The compression was made upon the femoral artery, where it passes over the pubic bone, for about eighty hours, by the members of the class, with the effect of causing considerable diminution of the tumor, and weakening the pulsations. The patient finding the pressure unbearable, the femoral artery was ligated, and the ligature came away on the eighteenth day.

This case should not be considered as one in which the treatment was thoroughly tried, since the pressure, made by so many persons, was very irregular. At the present time the tumor is rapidly diminishing and is perfectly solidified.—(*Medical and Surgical Reporter*, Nov. 17th, 1860.)

II.—*Wound of the Brachial Artery cured by Digital Compression.* By DR. BURY. (Gazette Médicale de Paris, No. 43, 1860.)

Digital compression need not necessarily be restricted to the treatment of aneurism and inflammation of the extremities, as proposed by M. Vanzetti, since it will be seen, from the case of Dr. Bury, that it is equally applicable in hemorrhage, even from a large artery. M. Marjolin has resorted to it successfully in three cases: one being an aneurism of the palmar arch; one a wound of the radial artery in a child; and in the third it was employed for one hour and a quarter, to arrest the bleeding consequent upon the operation of lithotomy. If it does not succeed, no bad result will follow, and recourse can afterward be had to the ligature.

A man, aged thirty-seven, received a blow upon the right arm, on the seventeenth of June, and on the following morning a physician found the limb enormously swollen, and of a violet color, due to the effusion of blood. He made two incisions over the swelling, which were followed by the escape of a large quantity of blood, after which the bleeding seemed to have stopped. In twenty-four hours, the dressings being soaked, he was taken to the Saumer Hospital, and on removing them the blood flowed by jets, and was restrained, but not completely, by compression. For several days compression was employed, aided by agaric and perchloride of iron, used externally and internally, but failed to answer the purpose, and on removing the clots, the blood escaped in such abundance as to leave no doubt of the brachial artery being wounded. The patient was much prostrated, and the condition of the arm and wound rendered it difficult to ligate the artery at the point of injury. The fear of mortification prevented Dr. Bury ligating the axillary artery, and he, therefore, determined to resort to digital compression. On the twenty-second of June, a resident and four sisters of the hospital commenced the treatment by compressing the upper portion of the brachial artery, and it was continued uninterruptedly for sixteen hours. At the end of forty-eight hours the hemorrhage had entirely ceased, but as a matter of precaution the pressure was continued some time longer. Under its influence the pain in the arm was much relieved and the swelling greatly diminished. The patient was discharged on the thirty-first of July, at which time the wounds of the arm had closed.

III.—*Intra-Uterine Dislocation of the Knee-Joint.* By J. YOUMANS, M.D. (Boston Med. and Surg. Journal, Oct. 25, 1860.)

Dr. Youmans met with an interesting case of intra-uterine dislocation of the left knee-joint, in 1859, the patella being smaller than the one of the opposite limb. The mother was conscious of having received an injury two weeks previous to her delivery, in consequence of overreaching while house cleaning. The child, at the time of the report, had perfect use of her limb.

Dr. Youmans relates the case in the following terms:—

"The patient, a laboring woman, under the medium size, was delivered, after a natural labor, of a full-sized, healthy female child, whose left knee-joint was entirely dislocated, so that the toes rested upon the anterior part of the thigh near the groin. As soon as I could leave the mother, I took hold of the limb and brought it to its natural position; but to my surprise, as soon as I relinquished my hold, it flew back, as with a spring, to its former position; it was immediately brought again to its place and held by the hands of the nurse, while I manufactured an apparatus for the occasion. Two pieces of whalebone, one on each side of the limb, were fastened together with three pieces of tape, one passing under the popliteal space, the other two in front, above and below the knee. This served a good purpose for keeping the leg in position, and also occupying but a small space, admitted of cleansing and dressing the child without removal. Some swelling and soreness occurred, and several weeks elapsed before the splints could with safety be removed."

IV.—*New Operation for the Radical Cure of Inguinal Hernia.* By D. HAYES AGNEW, M.D., Surgeon to the Philadelphia Hospital. (Medical and Surgical Reporter, Nov. 17, and Dec. 1, 1860.)

Two cases of inguinal hernia of long standing have been cured by Dr. Agnew's method, one occurring in his own practice, the other in that of Dr. Garretson. The instruments necessary for the operation consist, first, of two semi-cylinders of steel, three inches long and one inch and a quarter in circumference, which can be separated from each other by a screw in the handle of the instrument, and on the internal face of the lower blade of which are two parallel longitudinal grooves. Secondly, of a spear-pointed needle, slightly curved at the extremity, and supported on a bone handle. Third, of several stout needles from two to two and a half inches in length.

A portion of the scrotum is carried into the external abdominal ring, followed by the metal cylinder, and thus thrust up to the internal ring. The blades are now separated by means of the screw attached to its handles, and the long needle, armed with a thread of silver wire, is carried along one of the grooves to the upper end of the cylinder, and then made to pierce the exterior parietes of the inguinal canal; the thread is then removed from the needle, the latter withdrawn, and the other end of the wire passed through its eye, when it is passed along the other groove and made to emerge upon the surface of the skin, a short distance from the first. It is again unthreaded, and after the removal of the needle the two ends of the wire are drawn up tightly and twisted over a small roll of lint. This effectually holds up the plug of integument to the very summit of the canal, and as the silver thread manifests but little disposition to ulcerate out, it may be allowed to remain for a considerable time. The second and most important step of the operation consists in screwing the handles of the instrument completely together, thus separating the blades in the canal to their greatest possible extent, and then carrying across the canal, between the blades, four or five threads, at equal distances from each other. The first thread should be composed of silk, and be introduced as near the internal ring as possible; the remainder should be of silver, and the last one be close to the external ring. These transverse threads can be lodged in the canal with great accuracy by this method, the cord being protected from injury by the posterior blade of the instrument. The latter being withdrawn the patient is confined to his bed; the parts being protected by a compress and roller, and the wires should be removed so soon as the plastic exudation has bound the invaginated plug to the walls of the canal, which, in the cases operated upon, required twelve days.

The patient of Dr. Agnew was fifty-five years of age, who had an oblique inguinal hernia of seventeen years' standing; that of Dr. Garretson occurring in a lad, fourteen years of age, who had been affected with an oblique inguinal hernia, which had descended into the scrotum, since his second year. In both cases the parts remain perfectly invaginated and indurated, and the patients are walking about. The compress is still worn to favor firm consolidation.

V.—*New Osteoplastic Operation upon the Nose.* By PROFESSOR B. LANGENBECK. (Echo Médical, No. 7, 1860; Gazette Hebdomadaire, No. 40, 1860.)

From a paper on osteoplastic operations, by Professor Langenbeck, published in the *Deutsche Klinik*, No. 48, 1859, we extracted the details of an operation devised by the distinguished Berlin surgeon, for the removal of polyps affecting the nose and pharynx, (see this Journal, p. 702, 1860,) in which it was shown that a portion of bone when separated from its osseous connections could unite, provided it remained adherent by periosteum to the parent bone. In the same communication the author adverted to a case in which he had transplanted the pericranium upon the back of the nose, in order to replace the nasal bones, but in regard to the success of the attempt he entertained serious doubts. Dr. Dor has, however, followed up the case, the history of the first report having been incomplete, and has shown that the fears of Dr. Langenbeck were useless. The result of the operation being interesting, we give an abstract of the case at the present time.

A woman, forty years of age, had suffered for two years with ozæna and perforation of the palate, and complete destruction of the nasal bones and septum. The nose was in consequence sunken in, and the soft parts, which were healthy, were drawn up toward the forehead. The patient denied most positively that she had ever been affected with syphilis, although the disease could be traced to that source. At the time she had a purulent discharge from the mucous membrane of the pharynx, which yielded in four weeks to the iodide of potassium.

The operation was performed on the 17th of November, 1859, by penetrating the cavity of the nose, by an incision made from one wing to the other, thus separating the soft parts from the nasal apophysis, the frontal and superior maxillary bones. The point of the nose was then brought down toward the lip and the semilunar space thus made was filled up by a flap of a similar shape, removed from the forehead. The flap was composed not only of the skin, but also contained the periosteum, and the pedicle was attached near the internal angle of the right eye. The only difference between this and other rhinoplastic operations consisted in including in the flap of skin a portion of periosteum to an extent corresponding with the size of the lost bones. The after-dressing was confined to covering the wound with charpie, and introducing the same substance into the nose, which was, moreover, covered with a compress wet with cold water. On the fifth day the swelling was greater than is usual after such operations, and the wound had almost entirely united by the first intention. On the second of December, when Dr. Dor again saw the patient, the swelling had nearly disappeared, and the color of the skin was normal. There were at this time no signs of exfoliation of the denuded bone. On the sixth of February the bridge of the nose was perfectly firm, and the introduction of a sound into the nasal cavity gave the sensation of true bone. The internal surface was smooth and resistant, and the formation of new bone between the skin and periosteum appeared to have taken place. The cutaneous flap, which was at first excessively tumefied, had returned to its normal dimensions, and, under the microscope, osseous tissue, which did not differ from true bone, was observed. Granulations readily formed on the denuded portion of the frontal bone, a very small portion of which exfoliated.

VI.—*Clinical Report on Epithelial Cancer of the Lip.* By JONATHAN HUTCHINSON, M.D. (Medical Times and Gazette, Nos. 536, 537, 1860.)

This report embraces a statistical analysis of one hundred and twenty-seven cases of epithelioma of the lip, occurring in hospital practice, in all of which operations for the removal of the disease had been performed. The lower lip was affected in ninety per cent. of the cases, the upper in four per cent., and the angle of the mouth in six per cent. From the series, we find that women are subjects of the disease in the proportion of five to every hundred males, and when they are affected, it is most frequent in those who are in the habit of smoking. The average age of the patients was fifty-eight years, the extremes being twenty-eight and eighty-two years. In all excepting about twelve cases was the disease primary.

The results of the operations for the removal of cancer of the lip in one hundred and twenty-seven cases may be summed up as follows: Three patients died of erysipelas within ten days of the operation; in seven, the cancer returned in the wound; nine had a return of the disease in the cicatrix at different periods after the operation; in five the lymphatic glands became affected subsequently; three had the same disease on the opposite part of the lip; and one hundred and five are reported as having recovered from the operation, having left the hospital with sound cicatrixes. Inasmuch as most of the reports were made within a few months of the operation, a sufficiently long period had not elapsed to discover whether the disease had returned in a larger proportion of cases than above indicated.

VII.—*Clinical Report on Nineteen Cases of Epithelial Cancer of the Tongue.* By JONATHAN HUTCHINSON, M.D. (Medical Times and Gazette, Nos. 538, 539, 1860.)

Cancer of the tongue being very rapid in its progress, rarely calls for surgical interference, since the disease is usually very far advanced when the patient is brought under the notice of the surgeon. In all but two of the cases quoted in this report were operations performed, but it is probable that four times as many cases were treated in the hospital during the period to which the report refers.

The average age of the patients was fifty-four, the extremes being thirty-two and seventy-eight In one case the sex is not stated; of the remainder, twelve were males and six females. In two cases, on account of the advance of the disease, palliative measures were resorted to, one patient dying within five months of its commencement, the other within seven months. In seventeen cases the cancer was removed: by the knife in eight, by the ligature in five, and by the écraseur in four. Three of these died, two of pyæmia within seven weeks, and the other in about a month. Ten patients left the hospital with the wound healed, and in four the disease returned either before the wound had closed, or shortly after. Of the cases operated upon in which the duration of the disease is given, it had existed in one three months, in one four months, in one five months, in one eight months, in one a year, in one eighteen months, and in one two years.

In continuing this report, Dr. Hutchinson adds additional facts in regard to the clinical history of epithelioma of the tongue, and, in the *Medical Times and Gazette*, No. 541, 1860, gives a tabular statement of sixteen cases, furnished him by Dr. Humphry. As this contribution is especially valuable, on account of the history of the majority of the cases being complete, we reproduce it here :—

| No. | Age & Sex. | Nature and Duration of Ulcer, State of Glands, etc. | Treatment. | Result. |
|---|---|---|---|---|
| 1 | F.; 44. | Indurated ulcer at raphé; enlarged submaxillary glands. | Removed by ligature six months after commencement of disease. | Died 14 months after commencement of disease. |
| 2 | F.; 60. | Indurated ulcer at hinder part of right margin; glands not affected. | Palliative. | Died 18 months after com. of disease. |
| 3 | F.; 68. | Induration of right edge and adjacent part of floor of mouth; glands not affected. | Palliative. | Died 15 months after com. of disease. |
| 4 | F.; 23. | Indurated ulcer on the right edge of tongue near palate; glands at angle of jaw slightly enlarged in second year. | Palliative. | Died 3 years and 3 months after com. of disease. |
| 5 | M.; 52. | Superficially ulcerated and indurated right edge of tongue; no affection of glands. | Palliative. | |
| 6 | F.; 60. | Hinder part of right edge; no affection of glands. | Palliative. | Died 18 months from com. of disease. |
| 7 | F.; 54. | Deeply fissured ulcer, size of shilling, on middle of right edge; base indurated to some extent around; eight months' duration; no disease of glands. | Excised, Aug., 1848. | Quite well, March, 1857. |
| 8 | F.; 70. | Hinder edge of tongue and pillar of palate. | Palliative. | |
| 9 | M.; 66. | Middle of left edge; uneven ulcer on indurated base, size of half penny; four months' duration; no disease of glands. | Excised, March, 1849. | Disease returned as soon as the wound healed; died 9 mos. after operation. |
| 10 | F.; 40. | Flat, uneven ulcer on indurated base; middle of left edge of tongue; no disease of glands; three or four months. | Excised, Aug., 1852. | Disease returned in 6 months; died Feb., 1854. |
| 11 | F.; 64. | Right side of tongue; no disease of glands; was alive a year after commencement of disease. | Palliative. | |
| 12 | M.; 46. | Whole length of left side; five months; enlarged glands. | Palliative. | Died 15 months from com. of disease. |
| 13 | M.; 45. | Indurated fissured ulcer on right edge nearer tip than usual; six months; glands slightly enlarged. | Excised. | Well 6 months after operation. |
| 14 | M.; 61. | Indurated mass near tip, extending to subjacent tissue; three months; no disease of glands. | Palliative. | Died 20 months from com. of disease. |
| 15 | M.; 53. | Ulcerated warty growth on left edge; a few months; no disease of glands. | Excised, July, 1859. | Well now. |
| 16 | M.; 54. | Crooked indurated ulcer size of half-penny on left edge nearer tip than usual, caused by a bite nine months ago: no disease of glands. | Excised, Feb., 1859. | Well now. |

VIII.—*Case of Recto-Vesical Lithotomy, in which Bozeman's Button Suture was employed.* By J. F. NOYES, M.D. (Boston Medical and Surgical Journal, Nov. 29, 1860.)

A year since we reported a case of recto-vesical lithotomy, in which Dr. Bauer closed the wound with silver sutures, union taking place, by the first intention, in eight days. This favorable result induced Dr. Noyes to apply the wire for a similar purpose—with the addition, however, of the leaden shield—in the case of a man, thirty-nine years of age, who had suffered with symptoms of vesical calculus for three years. As he was much emaciated and prostrated, an effort was made to improve his general health, but nothing being gained by delay, the recto-vesical operation was performed on the 21st of October, 1859. The patient being under the influence of ether, was placed on his left side, with the thighs flexed and separated, and the rectum was dilated with the ordinary speculum. An incision was made through the central portion of the prostate, and enlarged bilaterally sufficiently to allow of the introduction of the index finger, when the stone, which was an inch and a half long by three-fourths of an inch in width, was removed by the forceps. The wound was closed by six silver sutures, and Bozeman's button was adjusted. The apparatus was removed on the twelfth day, and all the wound, excepting a small spot in the centre, was found to have united. A few applications of nitrate of silver had the effect of causing this spot to close by granulation, and at the end of three months the patient had gained about seventy pounds.

IX.—*Five Cases of Neurotomy for Painful Affections of the Limbs.* By REDFERN DAVIES, M.D. (Dublin Quarterly, Nov., 1860, p. 331.)

CASE I.—A laborer, aged fifty-two, sustained a compound fracture of the middle third of the tibia and lower third of the fibula, in consequence of a fall a distance of forty feet. In five months the limb was sound, as far as the fracture was concerned, but he suffered from a severe pain, which commenced during the process of union. His condition on the 20th of November, 1857, about thirteen months after his discharge from the hospital, was as follows: He was tortured with a persistent, gnawing pain, greatly aggravated by motion and by the touch, which commenced on the dorsal surface of the toes, extending over the foot, anterior surface of the ankle and leg as high as the original site of the tibial fracture. From this point it was felt in a line running to the interval between the fibula and edge of the gastrocnemius, and thence descended to the outer ankle and outside of the foot, joining with the pain on the dorsum. The limb was shrunken and flabby; the foot partially extended; the toes flexed, cold, and destitute of feeling. On embracing the leg with both hands and making firm pressure with the thumbs over the first intermuscular space on the anterior surface above the seat of fracture, the pain in the dorsum of the foot and front of the leg was much diminished, the other remaining unaffected. Dr. Davies accounted for the anterior pain by supposing the anterior tibial and musculo-cutaneous nerves to have been implicated by the tibial fracture; the sympathetic suffering between the tendo-Achillis and the fibula, and in the outer part of the ankle and foot being due to the communication between the communicans peronei and external saphenous nerve. To relieve the suffering, an inch of the popliteal nerve was removed, and on recovering from the effects of the anæsthetic, the pain was found to have disappeared, and the limb could be jolted. At the end of three months the progress of the case is thus described:—

"The limb, when in a state of rest, has the toes drawn downward; the foot turned inward, and the heel raised. All power of extension is completely gone; sensation is less acute than on the opposite side, but it is gradually improving; he also perceives himself that the dorsum of the foot and anterior surface of the leg are much colder than the posterior and plantar surfaces; and to the thermometer there is a difference of four degrees in the temperature of the part above and below the division of the nerve; a similar difference exists, likewise, between the temperature of the two legs. Since the operation, there has been no return of pain; and he has this day—February, 1858,—walked, with the aid of a slightly-raised heel to his shoe, a distance of six miles, in perfect ease and comfort."

CASE II.—A locksmith, aged sixty-seven, experienced a fracture of the middle third of the thigh-bone, three years ago, which, at the end of twelve weeks, had consolidated, with one inch of shortening. He has been unable ever since the accident, in consequence of the great pain, to put his foot to the floor, or bear any weight on it. Upon examining the limb, October 1st, 1858, the posterior muscles of the leg were found to be atrophied, rigid, and hard; the foot was extended, the toes flexed and apparently immovably fixed, all attempts to straighten them being attended with great suffering; the ankle-joint was free, but motion caused agonizing pain; and the temperature of the limb was five degrees lower than the sound one. In regard to the site of fracture, there was no deformity. Under the influence of chloroform the leg became natural, the muscles of the calf being soft, and the toes resuming their normal condition and being freely movable. As the effects of the anæsthetic passed off, the parts returned to their abnormal state. Under the influence of a freezing mixture, and during sleep, the same phenomena were witnessed. These indications led Dr. Davies to exsect about an inch of the posterior tibial nerve, the operation being performed in the lower third of the leg, as for the ligation of the posterior tibial artery. This was followed by immediate cessation of the pain and a normal condition of the toes. The thermometer indicated no difference in the temperature of the two legs. The patient progressed favorably, and the history of this case is brought up to October, 1860, when he could, with the aid of a high-heeled shoe, rendered necessary from the shortening produced by the fracture, walk about and work, standing about nine hours a day without experiencing the slightest pain or inconvenience.

CASE III.—A woman, fifty years of age, received a knife wound, in May, 1858, just below the pisiform bone, since which time she has suffered intense pain, shooting up to the shoulder, whenever the ring or little finger was touched or the forearm was jarred. They were frequently affected with painful twitchings, both during sleep and when awake. All the fingers were semi-flexed, and the last two were cold and clammy. Six months after the accident, half an inch of the ulnar nerve was removed above the pisiform bone, and the fingers could be immediately roughly handled without the production of pain, and they were also warmer than before the operation. Ever since this time she has been perfectly free from suffering, could use the limb almost as well as ever, the only difference being that she could not appreciate slight differences in textures by the touch, or pick up very small objects.

CASE IV.—A laborer, aged fifty-nine, sustained an injury to his left wrist-joint from a fall, in 1845, and five years subsequently his arm was amputated at the upper third. In removing the last ligature, on account of the patient's starting, it was twitched away, and pain instantaneously ensued in the stump. This gradually increased until it became constant; the end of the stump became excessively tender and was intolerable of the slightest pressure. In 1852, the skin was separated from the deeper tissues of the stump by a tenotome introduced subcutaneously. In 1853 and 1857 tumors were removed from the end of the stump, followed by an improvement in the condition of the patient and a relief from pain, which lasted, however, only about six months. In June, 1859, Dr. Davies removed about half an inch of the median, ulnar, and musculo-spiral nerves. This operation was followed for a brief space by good motion of the stump and cessation of pain, but a year subsequently the pains had returned with their usual force.

CASE V.—A sailor, thirty-two years of age, came under the care of Dr. Davies, in May, 1860, presenting the following symptoms: The left foot was cold and clammy and affected with equino-varus, the leg atrophied, and the toes contracted and rigid. He complained of constant pain in the great and second toe , extending along the back of the foot, up the leg as far as the tubercle of the tibia, and relieved by firm pressure made above. He had also pain all over the sole of the foot and heel, following the course of the posterior tibial nerve, up to the popliteal space. This pain he described as attacking him suddenly, lasting from one to twelve hours, and being similar to the shock of a galvanic battery. Six years previously, when on a cruise, he was the subject of yellow fever, and was sick for six months. This was followed by contraction of and pain in the toes, which obliged him to quit the service in 1859. The anterior tibial nerve was divided above the ankle and three-fourths of an inch removed, the result being relief in the big toe and the anterior portion of the leg. Two weeks subsequently, the posterior tibial nerve was dealt with in a similar manner, and with as happy a result, the patient being able to walk about and even strike his foot on the ground. During the operation, a thermometer was applied to the limb, and after the division of the nerve the temperature increased three degrees. The remaining equino-varus was relieved by the usual procedure.

